# Supplementary material for: From genomic signals to prediction tools: a critical feature analysis and rigorous benchmark for phage–host prediction
Source: Brief Bioinform. 2025 Nov 24;26(6):bbaf626. doi: 10.1093/bib/bbaf626 (PMC12641615; doi:10.1093/bib/bbaf626)
Supplement: Supplementary_bbaf626 [file supplementary_bbaf626.pdf]

Supplementary for "From genomic signals to prediction tools: a critical feature analysis and rigorous benchmark for virus-host prediction"

Jiayu Shang
Dept. of Information Engineering
Chinese University of Hong Kong
Kowloon, Hong Kong SAR, China

Cheng Peng
Dept. of Electrical Engineering
City University of Hong Kong
Kowloon, Hong Kong SAR, China

Jiaojiao Guan
Dept. of Electrical Engineering
City University of Hong Kong
Kowloon, Hong Kong SAR, China

Dehan Cai
Dept. of Electrical Engineering
City University of Hong Kong
Kowloon, Hong Kong SAR, China

Donglin Wang
Sch. of Environmental Science and Engineering
Shandong University
Qingdao, Shandong, China

Yanni Sun
Dept. of Electrical Engineering
City University of Hong Kong
Kowloon, Hong Kong SAR, China

Supplementary Note 1: Advantages and disadvantages of different virus-host prediction formulations

Data properties and imbalance

The two formulations contend with fundamentally different data distributions, particularly concerning class balance. The link prediction framework is inherently prone to extreme data imbalance. In a typical metagenome, a microbial community containing N viruses ($\{v_1, \ldots, v_N\}$), and M prokaryotes ($\{h_1, \ldots, h_M\}$), the number of true virus-host interactions is vastly smaller than the total number of possible pairings ($N \times M$). In the most extreme case, where each virus infects only one host, the ratio of positive links to negative (non-interacting) links is N to $N \times (M - 1)$. Given the immense number of viruses and microbes in environmental samples, this sparsity means that negative instances overwhelm positive ones, creating a significant hazard for model training and evaluation. A classifier can achieve high accuracy simply by predicting "no interaction" for all pairs, yet have no practical utility. The multi-class classification formulation mitigates this primary imbalance by reducing the prediction space from $N \times M$ potential pairs to just L host taxonomic classes. However, it still faces a different challenge: a long-tail distribution of class labels. Due to historical research biases and the natural abundance of certain microbes, a few taxonomic groups are heavily over-represented in the sequencing data, while the vast majority of taxa are rare (see Fig 5). This skewed distribution can lead to models that are highly proficient at predicting common hosts but perform poorly on under-represented or novel ones.

Feature representations

While not a rigid division, the two formulations naturally favor different types of feature extraction/processing. Link prediction models typically rely on pairwise features that represent direct evidence of a potential interaction between a specific virus (v_i) and a specific host (h_j). These features often include direct sequence homology between the virus and microbial genomes, matches between viral sequences and host-encoded CRISPR spacers, the presence of integrated prophages within a microbial genome, and similarities in tRNA gene repertoires, which can indicate host adaptation. In contrast, multi-class classification models predominantly use virus-centric features. The goal is to generate a feature embedding or "fingerprint" for each virus that is predictive of its host's taxonomy. These are often methods that capture intrinsic genomic properties, such as codon usage patterns, protein domain content or protein organization on the viral

genomes. The model then learns to propagate the host taxonomic labels from reference viruses with known hosts to the query viruses by comparing their similarity.

Nature and scope of predictions

The output and interpretation of predictions differ markedly between the two approaches. The link prediction formulation has the distinct advantage of providing explicit virus-host genome pairings. This high-resolution output is directly actionable for downstream analyses, such as identifying the hosts of viruses that carry antimicrobial resistance genes (ARGs) or auxiliary metabolic genes (AMGs). However, its primary drawback is the risk of a high false-positive rate. Because the model must evaluate all $N \times M$ candidate pairs, even a low error rate can generate a large number of incorrect predictions, requiring stringent probability thresholds or ranking to identify the most likely interactions. The multi-class classification formulation is more computationally efficient, as it makes only one prediction per virus. However, this comes with limitations. Standard classifiers assign a single, best-fitting label, which inherently struggles to predict polyvalent phages capable of infecting hosts across different species or genera. Furthermore, the prediction of a taxonomic label (e.g., *Enterobacteriaceae*) is less precise than identifying a specific host genome. A critical weakness is its "closed-set assumption": these models can only predict host taxa present in their pre-defined training set. They are unable to assign a host that belongs to a novel taxonomic group not seen during training, forcing an incorrect prediction from the available labels.

In essence, the choice between the link prediction and multi-class classification formulations represents a trade-off between precision and generality. The multi-class classification approach often achieves higher precision, particularly in scenarios where a query virus shares significant homology with viruses in a well-annotated reference database. It excels at rapidly assigning labels based on established patterns. Conversely, the link prediction framework is more generalizable. By evaluating direct evidence between any potential virus-host pair, it is better suited for the de novo discovery of novel interactions within complex metagenomic sequencing samples, where many viruses and hosts lack close relatives in curated databases. Neither formulation is universally superior; rather, they are suited to different research questions and data contexts. Ultimately, regardless of the chosen framework, the predictive power of any model is derived from its ability to extract meaningful biological signals from raw sequence data. These signals, transformed into quantitative features, serve as the foundational evidence for linking a virus to its host. In the following section, we provide an overview of the most prominent features employed in computational virus-host prediction.

Supplementary Note 2: Summary of the existing tools

Despite the increasing number of published computational methods for virus-host prediction, their practical adoption can be limited by significant barriers, particularly for researchers who may lack specialized computational skills. The utility of a prediction tool from an end-user standpoint can be evaluated based on three key criteria: ease of installation, quality of documentation, and workflow automation.

First, a straightforward installation process is crucial. The use of package managers like Conda can mitigate complex software dependency issues and compilation errors and does not require a root permission, which often presents the initial obstacle to use. Second, clear and comprehensive documentation is required. This includes detailed instructions on downloading and preparing necessary databases, executing the software with example commands, and providing a runnable test case so users can confirm the tool is functioning correctly. Finally, an automated workflow greatly improves usability. An ideal workflow allows the software to perform a complete analysis, from processing a standard FASTA input file to generating a final host prediction, without necessitating manual intervention for intermediate steps.

To assess the current state of accessibility, we downloaded and attempted to run each of the tools discussed in this review. All tests were conducted on a High-Performance Computing (HPC) cluster, an Oracle M8-8 Enterprise Server. The cluster is equipped with CPU nodes featuring both Intel Xeon and AMD EPYC processors with up to 1TB of memory, and GPU nodes containing a range of cards including the RTX 2080Ti, 4080, 4090, and Tesla A100. This hardware configuration was selected to ensure our evaluation covered the diverse computational environments commonly available in research laboratories. The outcome of this evaluation is summarized below:

Link prediction

The tools listed below follow the link prediction formulation and generally require both viral and microbial sequences as input:

WIsH (2017): This method utilizes alignment-free genomic signatures, specifically oligonucleotide frequencies, as its predictive feature. It employs a fixed-order markov model to calculate the probability that a viral sequence was generated from a given prokaryotic host genome [1]. Website: https://github.com/soedinglab/WIsH; Available.

VirHostMatcher (2017) : VirHostMatcher (VHM) uses k-mer frequencies as its primary feature. It predicts hosts by measuring the similarity between viral and potential host genomes using various distance metrics, including Euclidean distance, Manhattan distance, and the Jensen-Shannon divergence [2]. Website: https://github.com/jessieren/VirHostMatcher; Available.

PHP (2021): Relying on k-mer frequency profiles, PHP models the distribution of these features using a gaussian mixture model. This probabilistic framework is then used to assign a virus to its most likely host [3]. Website: https://github.com/congyulu-bioinfo/PHP; Available.

PredPHI (2021): This tool extracts proteomic features, including the abundance of amino acids and other chemical component information derived from protein sequences. These features serve as input for a convolutional neural network that performs the host classification [4]. Website: https://github.com/xialab-ahu/PredPHI; Unavailable (Can only be used to reproduce the experiments in the paper. The inputs are hard coded and lack guidelines of how to use the scripts. Cannot be run on the test data.)

PHIST (2022): The methodology of PHIST is based on quantifying the number of shared k-mers between a virus and potential host genomes. The host with the highest count of common k-mers is identified as the prediction [5]. Website: https://github.com/refresh-bio/PHIST; Available.

VirHostMatcher-Net (2022): VirHostMatcher-Net (VHM-Net) is an upgraded version of VHM integrates multiple lines of evidence within a Markov random field framework. Its features include CRISPR spacer matches, homology scores from BLASTN alignments, and the output scores from the WIsH prediction tool [6]. Website: https://github.com/WeiliWw/VirHostMatcher-Net; Available.

PHIAF (2022): This approach combines k-mer frequencies and molecular weight as input for its predictive model. It utilizes a convolutional neural network to learn from these combined features [7]. Website: https://github.com/BioMedicalBigDataMiningLab/PHIAF; Unavailable (Can only be used to reproduce the experiments in the paper. The inputs are hard coded. Cannot be run on the test data).

CHERRY (2022): This tool is an upgraded version of HostG that constructs a heterogeneous network using diverse biological data, including CRISPR arrays, homology search results, k-mer frequencies, and protein organization. It then employs a graph convolutional network encoder and a deep neural network decoder for prediction [8]. Website: https://github.com/KennthShang/PhaBOX/; Available

iPHoP (2023): Functioning as an integrated pipeline, iPHoP aggregates results from multiple evidence types. It combines direct features like CRISPR matches and homology searches with the outputs from existing tools such as WIsH, VirHostMatcher, and PHP to form a consolidated prediction [9]. Website: https://bitbucket.org/srouxjgi/iphop/src/main/; Available

PhageTB (2023): PhageTB utilizes features derived from CRISPR spacer alignments and homologous protein searches. This information is then processed by an ensemble model to enhance the robustness of its host predictions [10]. Website: https://github.com/raghavagps/phagetb; Unavailable (Unable to install, pip failed).

PHPGCA (2023): PHPGCA utilize the network architecture previously developed in CHERRY. However, it introduces a distinct framework by employing a light graph convolutional network as its core encoder for prediction [11]. Website: https://github.com/JunPeng-Zhong/PHPGCA; Unavailable (Can only be used to reproduce the experiments in the paper. The inputs are hard coded. Cannot be run on the test data).

DeepPBI-KG (2024): This method is based on homology search results identified between viral and host DNA and gene sequences. These homology features are then fed into a dense neural network for classification [12]. Website: https://github.com/Tongqing-Wei/DeepPBI-KG; Unavailable (The example can be run successfully. The inputs are hard coded, especially the first step in running integrate_seq.py. User needs to place the FASTA file in a fixed folder, but it is not declared in the guidelines. In addition, all scaffolds in different MAGs will be combined into one file leading to wrong mapping in the prediction).

VHIP (2024): VHIP combines two types of genomic features: k-mer frequencies and the difference in GC content between the virus and host. For prediction, it employs a gradient boosting classifier, a form of tree-based ensemble model [13]. Website: `https://github.com/DuhaimeLab/VHIP_analyses_Bastien_et_al_2023`; Unavailable (There are no guidelines describing how to use the code).

PB-LKS (2024): This method uses k-mer frequencies to link phages and hosts. The prediction is made by identifying the host whose k-mer profile has the largest correlation coefficient with the phage's profile, using this value as a direct indicator of the relationship [14]. Website: https://github.com/wanchunnie/PB-LKS; Available

PHPGAT (2025): PHPGAT leverages the network architecture previously developed in CHERRY. Then, it applies a more advanced graph attention network v2 model to more effectively learn node importance within the network for prediction [15]. Website: https://github.com/ZhaoZMer/PHPGAT; Unavailable (Unable to install, pip failed).

MI-RGC (2025): This tool integrates k-mer frequency data with a phage association network built using mutual information from sequencing data. It then applies a regional graph convolution model to this graph structure to predict hosts [16]. Website: https://github.com/Ankang-Wei/MI-RGC; Unavailable (Can only be used to reproduce the experiments in the paper. The inputs are hard coded. Cannot be run on the test data).

Multi-class classification

The tools listed below are multi-class classifier and only consider viral sequences as inputs:

HostPhinder (2016): This method relies on homology-based evidence. It performs a homology search to find related phages with known hosts in a reference database and determines the host for a query phage via a majority vote among the hosts of the identified references [17]. Website: https://github.com/julvi/HostPhinder. Unavailable (There are no guidelines describing how to use the code.)

HostG (2021): HostG constructs a knowledge graph based on shared protein clusters between viruses and prokaryotes. A graph convolutional network is then trained on this graph to learn relational features and predict the host [18]. Website: https://github.com/KennthShang/HostG. Unavailable (Unable to install, conda failed).

RaFAH (2021): RaFAH first generates protein clusters using MMseqs2 and builds profile hidden markov models (HMM) from them. The resulting HMM alignment outputs are then used as features for a multi-class random forest model to perform host prediction [19]. [18]. Website: https://sourceforge.net/projects/rafah/files/RaFAH_v0.3_Files/. Available.

VPF-Class (2021): This framework predicts viral hosts by leveraging a database of classified Viral Protein Families (VPFs). It automates host prediction by assigning the proteins from a query viral contig to the pre-classified VPFs. The host is then inferred based on the known host information associated with the identified protein families, providing predictions typically at the genus level [20]. Website: https://github.com/biocom-uib/vpf-tools. Unavailable (Installation requires running as root, which is often impossible and dangerous on HPC).

DeepHost (2022): This tool encodes genomic sequences of various lengths into three-dimensional matrices using their k-mer features. A convolutional neural network is then trained on these matrix representations to perform multi-class host classification [21]. Website: https://github.com/deepomicslab/DeepHost. Available.

HoPhage (2022): HoPhage employs a hybrid approach by first constructing a markov chain model from the coding sequences of candidate hosts. The likelihood scores from this model are then integrated with the output of a separate deep learning model to generate a final prediction [22]. Website: https://github.com/jie-tan/HoPhage. Unavailable (The download link of the scripts cannot be accessed).

vHULK (2022): The predictive features for vHULK are derived from protein profile alignment results of a query phage against the pVOGs (prokaryotic Virus Orthologous Groups) database. This alignment profile serves as the direct input for classifying the phage into a host taxon [23]. Website: https://github.com/LaboratorioBioinformatica/vHULK. Available.

PHERI (2023): PHERI uses multiple binary decision tree classifiers to select host-specific protein clusters from reference phages. A query phage is then classified by aligning its proteins against these identified clusters to assign the most appropriate host label [24]. Website: https://github.com/andynet/pheri. Available.

PHIEmbed (2023): This method focuses on receptor-binding proteins (RBPs), generating feature embeddings for them using the pre-trained large protein language model. A random forest classifier then uses these embeddings as input to predict the host [25]. Website: https://github.com/bioinfodlsu/phage-host-prediction. Unavailable (Can only be used to reproduce the experiments in the paper. Requires receptor-binding proteins as inputs. However, there is no integrated receptor-binding proteins detection step in the code nor a suggested way to detect receptor-binding proteins in the guidelines. Cannot be run on the test data).

EvoMIL (2024): EvoMIL operates at the protein level by using a pre-trained large protein language model (ESM) to generate feature representations of viral proteins. It then applies an attention-based multiple instance learning

framework to these features for host prediction [26]. Website: https://github.com/liudan111/EvoMIL. Unavailable (Can only be used to reproduce the experiments in the paper. The inputs are hard coded. Cannot be run on the test data).

PHIStruct (2025): PHIStruct is an upgraded version of PHIEmbed that leverages protein structural information by using a structure-aware protein language model to generate 3D structure embeddings of receptor-binding proteins. These structural features are subsequently classified using a multilayer perceptron with two hidden layers [27]. Website: https://github.com/bioinfodlsu/PHIStruct. Unavailable (Can only be used to reproduce the experiments in the paper. Require 3D structure of receptor-binding proteins as inputs (pdb file). Same issues as the PHIEmbed. Cannot be run on the test data).

Table 1: Practical recommendations for virus-host prediction.

Research Goal	Input Data	Primary Consideration	Recommended Tool(s)
High-accuracy host identification (Genus/Family level)	Viral sequences only	Highest accuracy	RaFAH, CHERRY
High-accuracy host identification (Species level)	Viral sequences only	Highest accuracy	CHERRY
De novo host discovery in a new environment	Viral contigs + Host MAGs	Robustness across contexts	PHIST, CHERRY
Maximize prediction rate (get a prediction for every virus)	Viral sequences or Virus + MAGs	100% prediction rate	PHP, DeepHost
Maximize confidence (for validating a critical interaction)	Viral contigs + Host MAGs	Highest possible accuracy	**Ensemble: "Joint + Consensus" of CHERRY, PHIST, iPHoP**
Large-scale analysis with limited computational resources	Viral contigs	Speed and efficiency	PHIST, PHP, DeepHost, CHERRY

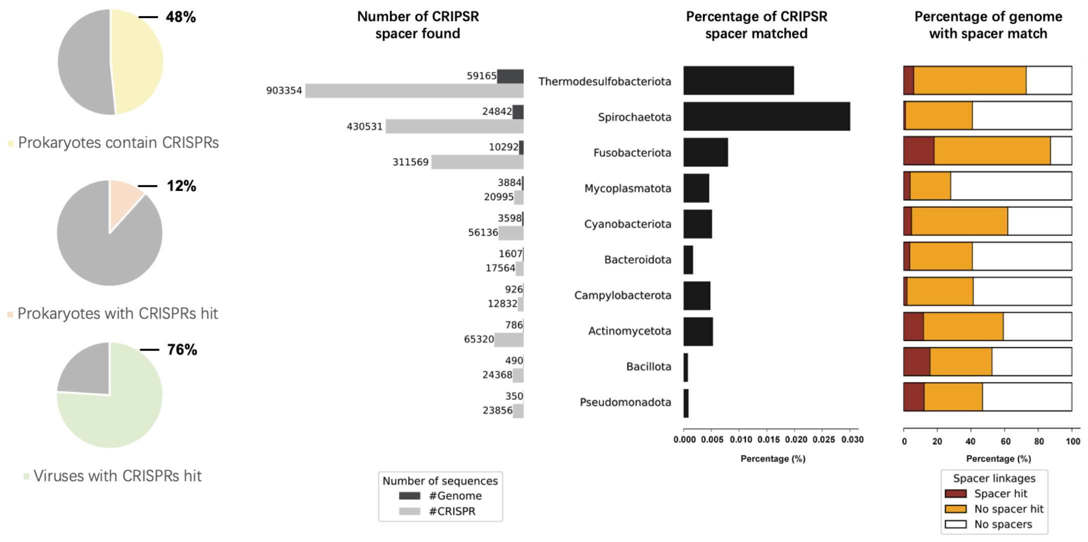

Figure 1: CRISPR-Cas spacer analysis reveals widespread but heterogeneous virus-host linkages. The figure illustrates virus-host interactions as determined by aligning 4,698 viral genomes against 2,005,489 CRISPR spacers from 110,988 prokaryotic genomes. The pie charts (left) show an overview of CRISPR-based virus-host linkage prevalence. The bar plot (right) shows a comparative analysis across the ten prokaryotic phyla with the most identified CRISPR spacers.

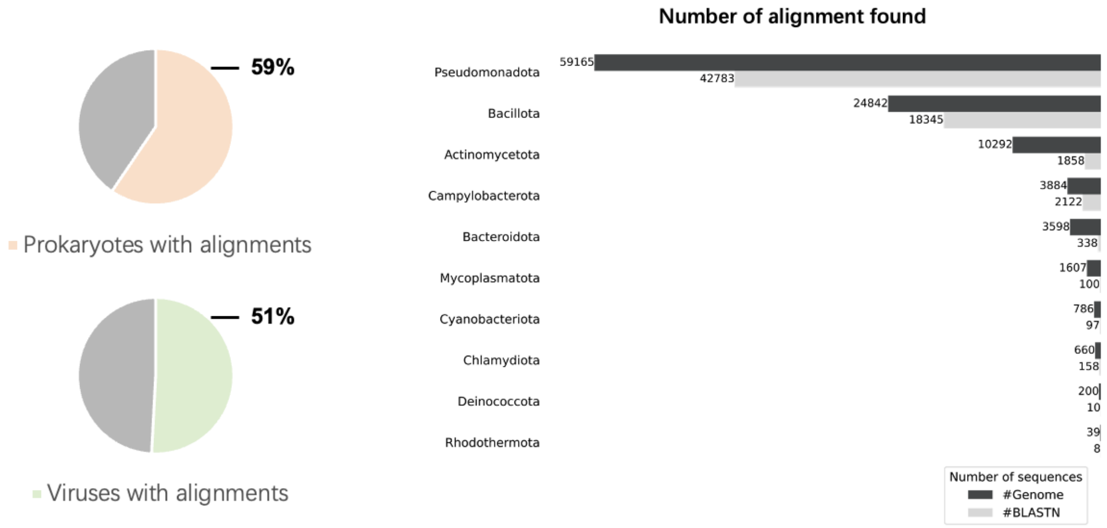

Figure 2: Virus-host linkages identified by direct sequence alignment reveal a distinct interaction landscape. The figure shows virus-host connections identified by BLASTN sequence alignments between the 4,698 viral and 110,988 prokaryotic genomes. The pie charts (left) illustrate the overall proportion of genomes with significant alignments. The bar chart (right) shows the number of alignments found within the ten prokaryotic phyla with the most genomes.

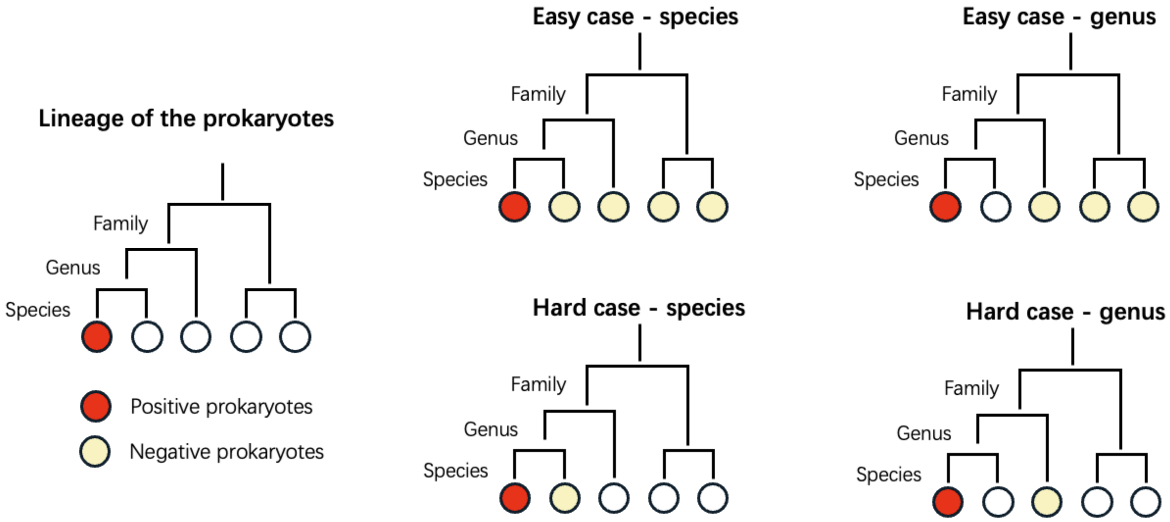

Figure 3: Schematic illustration of negative sampling strategies for k-mer frequency comparison based on taxonomy tree. The ’easy case’ sampling strategy: negative examples are selected from prokaryotes that are in different taxa from the given host. For instance, the negative prokaryotes belong to a different species or genus than the host. The ’hard case’ sampling strategy: negative examples are selected with a constraint on the taxonomy rank of the prokaryotes. For example, the negative prokaryotes are different species but belong to the same genus as the positive host.

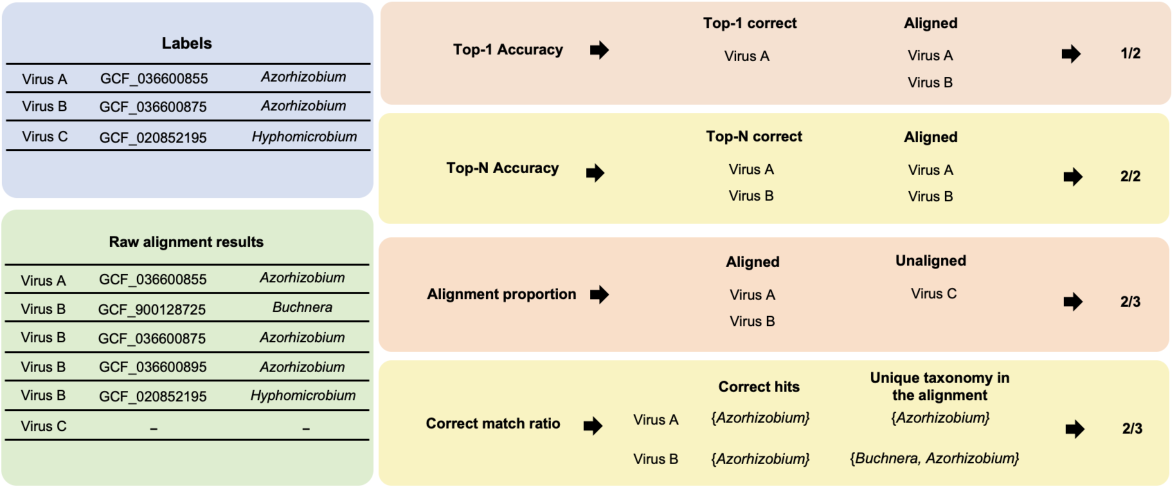

Figure 4: Metrics for evaluating the performance of CRISPR spacer and prophage matches. The figure illustrates the four statistical metrics used to evaluate the accuracy and coverage of CRISPR-based and alignment-based predictions. The calculations are demonstrated using a simplified example with three viruses (A, B, C). The ground truth interactions ("Labels") are shown in the top-left panel, while the hypothetical prediction outcomes ("Raw alignment results") are in the bottom-left panel.

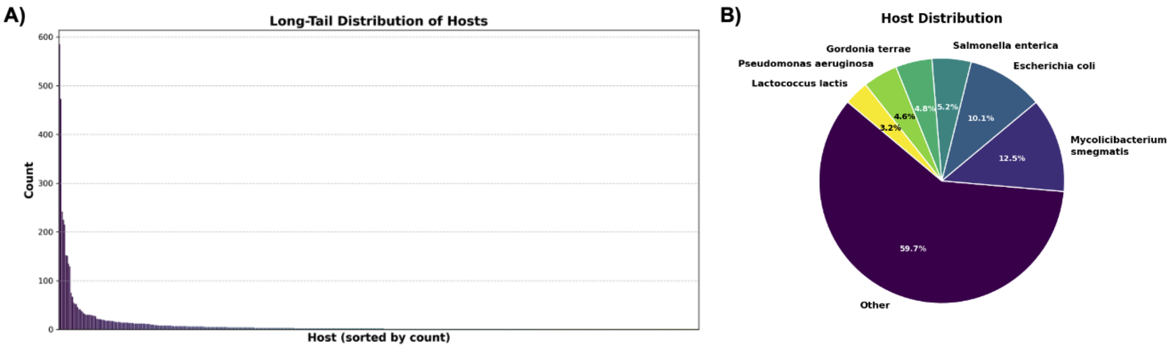

Figure 5: Host distribution in the RefSeq-VHDB dataset. The dataset contains 4,698 viruses linked to 498 distinct host species and exhibits a significant data imbalance. A) The bar chart illustrates the long-tail distribution of hosts, where a few species are associated with many viruses, while most are linked to very few. Notably, 245 species are represented by only a single virus. B) The pie chart shows the relative abundance of the most frequent host species. The top three hosts—Mycolicibacterium smegmatis, Escherichia coli, and Salmonella enterica—account for a combined 27.8% of all entries, highlighting the skewed nature of the dataset.

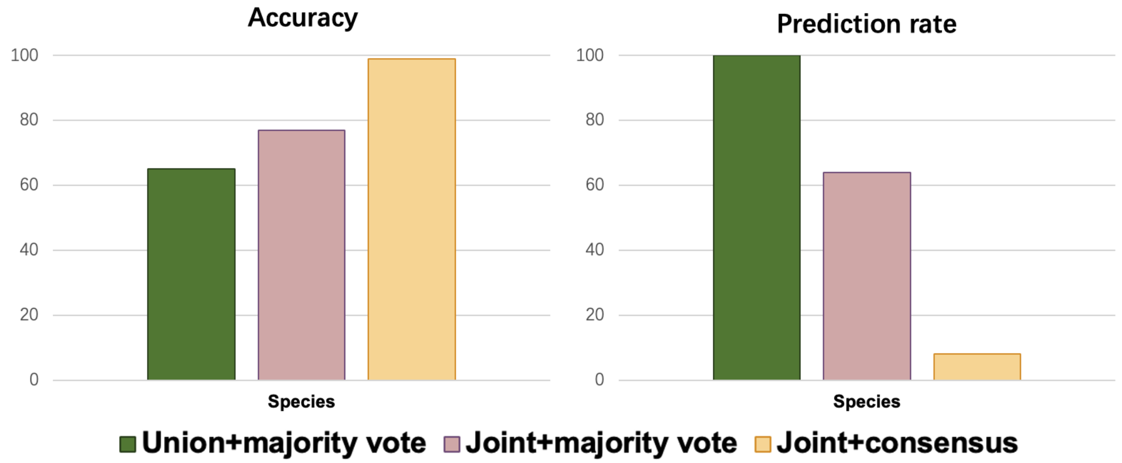

Figure 6: Performance of three ensemble strategies for host prediction on the RefSeq-VHDB benchmark. The figure shows (left) species-level accuracy and (right) the corresponding prediction rate for each strategy. The strategies involve combining the outputs of the top-performing individual tools. The 'joint + consensus' method achieves the highest accuracy (99%) but at a significant cost to the prediction rate (10%), illustrating a key trade-off between precision and coverage.

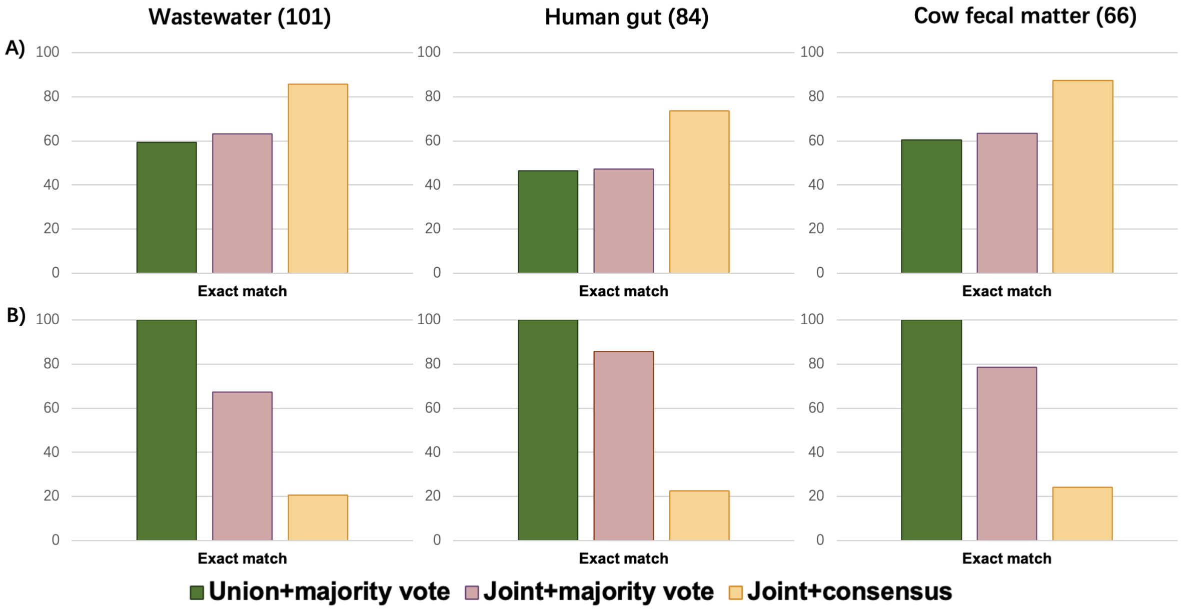

Figure 7: Performance of ensemble strategies across three distinct metagenomic datasets from the MetaHiC-VHDB benchmark. The datasets represent (left to right) Wastewater (101 pairs), Human gut (84 pairs), and Cow fecal matter (66 pairs). A) Exact match accuracy for the three combination methods. B) Corresponding prediction rate for each method. The results consistently demonstrate that the 'joint + consensus' approach achieves the highest accuracy in all environments, but at the cost of a reduced prediction rate. This reinforces that the fundamental trade-off between accuracy and coverage holds for complex metagenomic data.

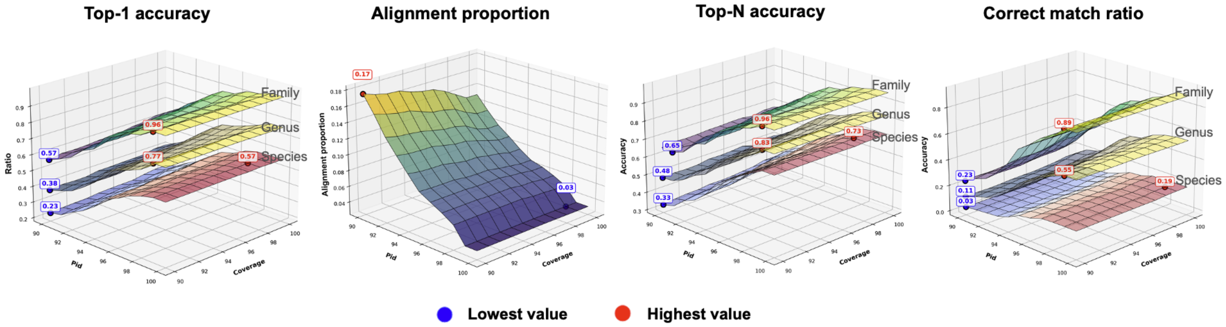

Figure 8: Surface plots to illustrate the utility of tRNA searching. We obtained 7,709,234 tRNA genes derived from the prokaryotic genomes using ARAGORN [?]. Then, NCBI BLAST+ v2.16 was employed to align viral genomes to the tRNA genes database. Top-1 accuracy: accuracy summarized on the best hit (the hit with the best alignment score). Performance is evaluated at the Species, Genus, and Family taxonomic levels, based on key alignment parameters: percent identity (Pid) and coverage. The lowest and highest values on each surface are highlighted with blue and red dots, respectively. The four metrics evaluated are: Top-N accuracy: accuracy summarized on all the alignment hits. Alignment proportion: the fraction of viruses with at least one alignment. Correct match ratio: a proportion of the correct hits in the alignment results, estimating the errors introduced by using Top-N accuracy.

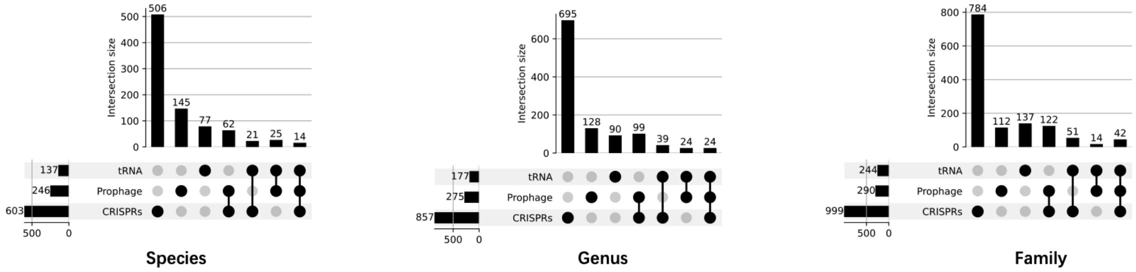

Figure 9: Overlap of correct virus-host predictions from CRISPR spacer analysis, prophage homology searching, and tRNA searching. The UpSet plots show the number of correct predictions unique to each method (single dots for prophage unique and CRISPRs unique) and the number shared by methods (connected dots) across the three taxonomic ranks. The horizontal bars indicate the total number of correct predictions for each method.

*

References

[1] Clovis Galiez, Matthias Siebert, François Enault, Jonathan Vincent, and Johannes Söding. Wish: who is the host? predicting prokaryotic hosts from metagenomic phage contigs. *Bioinformatics*, 33(19):3113–3114, 2017.

[2] Nathan A Ahlgren, Jie Ren, Yang Young Lu, Jed A Fuhrman, and Fengzhu Sun. Alignment-free oligonucleotide frequency dissimilarity measure improves prediction of hosts from metagenomically-derived viral sequences. *Nucleic acids research*, 45(1):39–53, 2017.

[3] Congyu Lu, Zheng Zhang, Zena Cai, Zhaozhong Zhu, Ye Qiu, Aiping Wu, Taijiao Jiang, Heping Zheng, and Yousong Peng. Prokaryotic virus host predictor: a gaussian model for host prediction of prokaryotic viruses in metagenomics. *BMC biology*, 19(1):5, 2021.

[4] Menglu Li, Yanan Wang, Fuyi Li, Yun Zhao, Mengya Liu, Sijia Zhang, Yannan Bin, A Ian Smith, Geoffrey I Webb, Jian Li, et al. A deep learning-based method for identification of bacteriophage-host interaction. *IEEE/ACM transactions on computational biology and bioinformatics*, 18(5):1801–1810, 2020.

[5] Andrzej Zielezinski, Sebastian Deorowicz, and Adam Gudyś. Phist: fast and accurate prediction of prokaryotic hosts from metagenomic viral sequences. *Bioinformatics*, 38(5):1447–1449, 2022.

[6] Weili Wang, Jie Ren, Kujin Tang, Emily Dart, Julio Cesar Ignacio-Espinoza, Jed A Fuhrman, Jonathan Braun, Fengzhu Sun, and Nathan A Ahlgren. A network-based integrated framework for predicting virus–prokaryote interactions. *NAR genomics and bioinformatics*, 2(2):lqaa044, 2020.

[7] Menglu Li and Wen Zhang. Phiaf: prediction of phage-host interactions with gan-based data augmentation and sequence-based feature fusion. *Briefings in Bioinformatics*, 23(1):bbab348, 2022.

[8] Jiayu Shang and Yanni Sun. Cherry: a computational method for accurate prediction of virus–prokaryotic interactions using a graph encoder–decoder model. *Briefings in Bioinformatics*, 23(5), 2022.

[9] Simon Roux, Antonio Pedro Camargo, Felipe H Coutinho, Shareef M Dabdoub, Bas E Dutilh, Stephen Nayfach, and Andrew Tritt. iphop: An integrated machine learning framework to maximize host prediction for metagenome-derived viruses of archaea and bacteria. *PLoS biology*, 21(4):e3002083, 2023.

[10] Suchet Aggarwal, Anjali Dhall, Sumeet Patiyal, Shubham Choudhury, Akanksha Arora, and Gajendra PS Raghava. An ensemble method for prediction of phage-based therapy against bacterial infections. *Frontiers in Microbiology*, 14:1148579, 2023.

[11] Zhi-Hua Du, Jun-Peng Zhong, Yun Liu, and Jian-Qiang Li. Prokaryotic virus host prediction with graph contrastive augmentaion. *PLoS Computational Biology*, 19(12):e1011671, 2023.

[12] Tongqing Wei, Chenqi Lu, Hanxiao Du, Qianru Yang, Xin Qi, Yankun Liu, Yi Zhang, Chen Chen, Yutong Li, Yuanhao Tang, et al. Deeppbi-kg: a deep learning method for the prediction of phage-bacteria interactions based on key genes. *Briefings in Bioinformatics*, 25(6):bbae484, 2024.

[13] G Eric Bastien, Rachel N Cable, Cecelia Batterbee, AJ Wing, Luis Zaman, and Melissa B Duhaime. Virus-host interactions predictor (vhip): Machine learning approach to resolve microbial virus-host interaction networks. *PLoS computational biology*, 20(9):e1011649, 2024.

[14] Jingxuan Qiu, Wanchun Nie, Hao Ding, Jia Dai, Yiwen Wei, Dezhi Li, Yuxi Zhang, Junting Xie, Xinxin Tian, Nannan Wu, et al. Pb-lks: a python package for predicting phage–bacteria interaction through local k-mer strategy. *Briefings in Bioinformatics*, 25(2), 2024.

[15] Fu Liu, Zhimiao Zhao, and Yun Liu. Phpgat: predicting phage hosts based on multimodal heterogeneous knowledge graph with graph attention network. *Briefings in Bioinformatics*, 26(1):bbaf017, 2025.

[16] Ankang Wei, Zhen Xiao, Lingling Fu, Weizhong Zhao, and Xingpeng Jiang. Predicting phage–host interactions via feature augmentation and regional graph convolution. *Briefings in bioinformatics*, 26(1), 2024.

[17] Julia Villarroel, Kortine Annina Kleinheinz, Vanessa Isabell Jurtz, Henrike Zschach, Ole Lund, Morten Nielsen, and Mette Voldby Larsen. Hostphinder: a phage host prediction tool. *Viruses*, 8(5):116, 2016.

[18] Jiayu Shang and Yanni Sun. Predicting the hosts of prokaryotic viruses using gcn-based semi-supervised learning. *BMC biology*, 19(1):250, 2021.

[19] Felipe Hernandes Coutinho, Asier Zaragoza-Solas, Mario López-Pérez, Jakub Barylski, Andrzej Zielezinski, Bas E Dutilh, Robert Edwards, and Francisco Rodriguez-Valera. Rafah: Host prediction for viruses of bacteria and archaea based on protein content. *Patterns*, 2(7), 2021.

[20] Joan Carles Pons, David Paez-Espino, Gabriel Riera, Natalia Ivanova, Nikos C Kyrpides, and Mercè Llabrés. Vpf-class: taxonomic assignment and host prediction of uncultivated viruses based on viral protein families. *Bioinformatics*, 37(13):1805–1813, 2021.

[21] Wang Ruohan, Zhang Xianglilan, Wang Jianping, and LI Shuai Cheng. Deephost: phage host prediction with convolutional neural network. *Briefings in Bioinformatics*, 23(1), 2022.

[22] Jie Tan, Zhencheng Fang, Shufang Wu, Qian Guo, Xiaoqing Jiang, and Huaiqiu Zhu. Hophage: an ab initio tool for identifying hosts of phage fragments from metaviromes. *Bioinformatics*, 38(2):543–545, 2022.

[23] Deyvid Amgarten, Bruno Koshin Vázquez Iha, Carlos Morais Piroupo, Aline Maria da Silva, and João Carlos Setubal. vhulk, a new tool for bacteriophage host prediction based on annotated genomic features and neural networks. *Phage*, 3(4):204–212, 2022.

[24] Andrej Baláž, Michal Kajsik, Jaroslav Budiš, Tomáš Szemes, and Ján Turňa. Pheri—phage host exploration pipeline. *Microorganisms*, 11(6):1398, 2023.

[25] Mark Edward M Gonzales, Jennifer C Ureta, and Anish MS Shrestha. Protein embeddings improve phage-host interaction prediction. *PloS One*, 18(7):e0289030, 2023.

[26] Dan Liu, Francesca Young, Kieran D Lamb, David L Robertson, and Ke Yuan. Prediction of virus-host associations using protein language models and multiple instance learning. *PLOS Computational Biology*, 20(11):e1012597, 2024.

[27] Mark Edward M Gonzales, Jennifer C Ureta, and Anish MS Shrestha. Phistruct: improving phage–host interaction prediction at low sequence similarity settings using structure-aware protein embeddings. *Bioinformatics*, 41(1):btaf016, 2025.
